# Affixation patterns in native language and sequence processing by statistical learning mechanisms

**DOI:** 10.1017/ehs.2025.6

**Published:** 2025-02-14

**Authors:** Mikhail Ordin

**Affiliations:** 1 Laboratory of Language, Metacognition and Decision-Making, Coimbra Institute for Biomedical Imaging, Universidade de Coimbra, Portugal; 2 Faculty of Medicine, Universidade de Coimbra, Portugal

**Keywords:** suffixing bias, statistical learning, morphological typology, Suffix Effect, serial recall

## Abstract

The suffixing bias (the tendency to exploit suffixes more often than prefixes to express grammatical meanings) in world’s languages was identified a century ago, yet we still lack a clear account for why it emerged, namely, whether the bias emerged because general cognitive mechanisms shape languages to be more easily processed by available cognitive machinery, or the bias is speech-specific and is determined by domain-specific mechanisms. We used statistical learning (SL) experiments to compare processing of suffixed and prefixed sequences on linguistic and non-linguistic material. SL is not speech-specific, and we observed the suffixing preference only on linguistic material, suggesting its language-specific origin. Moreover, morphological properties of native languages (existence of grammatical prefixes) modulate suffixing preferences in SL experiments only on linguistic material, suggesting limited cross-domain transfer.

## Introduction

Appending an affix to the word stem is one of the most frequently exploited means to express grammatical meaning (e.g., tense-aspect, number, case, person, interrogation, subordination). An affix can be appended before the stem (i.e., prefix), after the stem (i.e., suffix), embedded within the stem (i.e., infix), or adding morphemes consisting of two parts, with one part preceding the root, and the other part following the root (i.e., circumfix)^i^. Other morphological processes stem form diachronic processes, e.g., (e.g., run-ran; mouse-mice) or mergers of two paradigms (go-went-gone, with go-gone pair from a verb *gān*, and went − past tense stemming from the verb *wend*). In this study, however, we zoom on basic affixation strategies: suffixation and prefixation.

Across the world’s languages, suffixes are used substantially more frequently than prefixes to express grammatical meaning (and infixes are exceptionally rare compared to the other two types of affixes). While linguists have identified a clear preference for suffixing in world’s languages (Sapir, 1921; Greenberg, 1957; Cutler et al., 1985; Dryer, 2005; Hawkins & Gilligan, 1988), the distinction is not strictly categorical, with some languages expressing grammatical meaning by both suffixes and prefixes (e.g., Basque, Irish Gaelic, etc.). In the World Atlas of Language Structures (WALS), Dryer (2005) classified languages on a spectrum from strongly suffixing to strongly prefixing, and the number of the former is 4.5 times larger than the latter, recapitulating a strong skew towards right-hand branching across world’s languages in syntax (Hawkins, 1983; Antinucci et al., 1979; Grosu & Thompson, 1977).

There are competing theories about the origin of this bias. A large body of literature has shown that language structures are determined by general cognitive constraints on auditory perception (Blevins, 2004; Neath, 1993; Macintosh, 1975; Repp, 1992), learning (Croft, 2001; Kersten et al., 1998; Hall, 1991), memory (Gibson, 2000), psychological resistance to fusing prefixing material in favor of fusing suffixing material (Enrique-Arias, 2022). These constraints define domain-general cognitive mechanisms, which act to select those variants of language code that are more easily processed by existing cognitive mechanisms. The selected variants are modified and passed on to the next generations by means of social learning and cultural evolution (Christiansen & Chater, 2001; Dienes et al., 1999; Lewis et al., 2006; Saygin et al., 2003; Smith et al., 2002). The general cognitive mechanisms evolved for processing the non-linguistic environment under pressure from natural selection and available neural and cognitive resources, i.e., constraints on learning, perception, memory, attention, as well as anatomical constraints on articulation (Christiansen & Chater, 2001; Ordin et al., 2021). Within this framework, domain-general processes that make the beginning of the auditory sequences more salient and therefore more easily memorized and recalled might have resulted in suffixing bias in languages. Anatomical speech production machinery makes the onsets of speech sequences more salient, contributing to perceptual salience. For example, pitch resetting after inhalations marks the left edges of the discrete speech sequences (“left” and “right” are used in temporal, not in spatial aspect, since in the auditory modality segmental information is unfolding temporarily, not spatially). At the onsets of constituents, the phonetic contrasts between voiced and voiceless segments are preserved more easily than at the end of the constituents, directing attention to the left edges.

Alternatively, it can be argued that suffixes can be more easily processed by the cognitive machinery that is tuned *specifically for speech processing*, i.e., this bias is speech-specific and not domain-general. For example, the interference of grammatical prefixes with lexical access could explain the skew towards using suffixes over prefixes across world’s languages (Clark, 1991; Hawkins & Cutler, 1988; Cutler et al., 2009). The beginning of the word is more important for lexical access than the end of the word, because the pool of potential word candidates becomes increasingly narrower as more and more segmental information is becomes available (Erdeljac & Mildner, 1999; Marslen-Wilson, 1987; Rodd, 2004). Therefore, left-most segments are most critical for the word activation, and variation at the left edge of the word impedes word recognition.

This account in terms of lexical access also fits with evidence from connectionist modeling, which demonstrates how constraints on memory and computation efficiency lead to increasing computational demands as a function of sequence length, hence processing the end of the sequence is more difficult than the beginning of the sequence. It is thus preferable to place information that is less relevant for lexical access towards the end of the sequence. Gasser (1994), using connectionist modeling, showed that suffixed words are more easily processed compared to prefixed words. In his computational simulations using the connectionist approach, the model accepted stimuli phoneme by phoneme and used backpropagation learning algorithm to detect stems and morphemes in words with suffixes (e.g., vibuni − vibuna); prefixes (e.g., ivibun − avibun); infixes (e.g., vikbun − vinbun); circumfixes (ivibuni − avibuna); mutations (e.g., vibun –viban); and deletions (e.g., vibun − vibu). The model itself was physiologically motivated (based on the physiological properties of the signal propagation in neural networks), and it identified the stems of suffixed words much better than those of prefixed words. This is an emergent approach to suffixing bias, which also draws on common physiological principles of information processing.

The discussion about the origin of the suffixing bias is ongoing. Experiments by Hupp, Sloutky and Culicover (2014) showed that native speakers of English (a strongly suffixing language) exhibit a preference for language and non-language sequences with variable endings (i.e., suffixes). The authors advocated a domain-general origin of suffix preferences, which potentially emerged from cognitive processes outside the language domain and was transferred to language (or ended up shaping languages). However, it could be argued that the flexible nature of the general cognitive mechanisms underlying suffixing preference that is promoted by Hupp et al. (2014) allows for the transfer of an essential bias to non-language domain. The plausibility and possibility of this interpretation was explored by Martin and Culberton (2020), who demonstrated that speakers of a strongly prefixing Bantu language exhibit different preferences in similarity judgement task both on linguistic and non-linguistic material, and their responses were opposite to those of English native speakers. This finding agrees with some studies showing that exposure to certain regularities in speech can influence how similar regularities are processed in non-speech sequences (Marcus et al., 2007).

The debate on the origin of any typological bias is difficult to resolve. If the suffixing preference is defined by the general cognitive machinery, it could still be reversed by experience with prefixation in the native language. Prefixation might emerge in particular languages by social learning, cultural evolution, and random fluctuations in diachronic development. Once prefixation is established, it can spread across a linguistic population because people try to adapt to cultural norms. Efficient processing of speech is a cornerstone of human cognition, adapting general cognitive machinery for a better processing of new typological properties. As the general cognitive machinery underlies processing of non-linguistic stimuli as well, new properties of the linguistic code could feedback on general cognitive mechanisms and impact the cognitive constraints and preferences, even if they are not defined by such constraints and mechanisms at the time they emerged.

Despite this challenge, the primary objective of the current study is to further address the question of whether suffixing bias is speech-specific, or whether it stems from general cognitive mechanisms that (1) are recycled for speech processing; and (2) shape the language code to be more easily processable by pre-existing cognitive machinery. The experiment was conducted with monolingual speakers of Spanish (a strongly suffixing language) and Basque-Spanish bilinguals (Basque uses both suffixes and prefixes to express grammatical meanings). Such Basque-Spanish bilinguals have more experience with linguistic prefixes compared to monolinguals.

We used an artificial language learning paradigm (Saffran et al., 1996) to study how adding a prefix or suffix to the recurrent stem-like constituents will interfere with learning and recognition of these constituents by statistical learning mechanisms. Statistical learning (SL) is a set of evolutionarily ancient cognitive abilities for processing sequential environmental stimuli (Conway, 2020) that are shared by taxonomically different species (Kikuchi et al., 2018; Milne et al., 2018). Ordin et al (2020; 2021) have suggested that, in the auditory modality, SL mechanisms evolved to detect breaks in statistical regularities within continuous environmental sensory inputs, that is, within a flow of statistical cues. Such breeches of statistical regularities in the flow of environmental stimuli correspond to rapid changes in the environment, which require behavioural response. In natural speech, such breaks often correspond to the beginning of linguistic constituents such as words or phrases, which allows for recycling SL for speech processing to detect discrete constituents in a continuous acoustic stream. This turns on a cascade of other cognitive processes related to extraction of the discrete constituents from a continuous sensory input, memorization (committing of these constituents to memory), categorization of these constituents into (grammatical) classes, semantic mapping, etc. If we see that suffixed sequences are more easily detected and recognized than prefixed sequences, and this preference is stronger or exclusive on linguistic material across two populations, we will be able to argue for a language-specific origin (i.e., specific to language faculty) of the suffixing bias. If, on the other hand, the suffixing advantage turns out to be stronger on non-linguistic than on linguistic material, it would be in line with the suffixing bias in world’s languages being shaped by general cognitive constraints and mechanisms. An effect of native language can be observed by looking at differences in the strength of suffixing preference in Spanish and Basque populations (Spanish is a strongly suffixing language, and Basque has both grammatical affixes and prefixes − a more detailed justification for the language choice is in the Methods section).

## Method

We used a statistical learning paradigm, when participants first listen to a continuous acoustic stream with embedded recurrent sequences (familiarization speech stream), and during a post-familiarization recognition test they need to listen to short sequences and report whether this sequence is a word (a recurrent sequence listed from the familiarization speech stream) from the artificial language they listened to or a not (a foil composed of the same sounds as recurrent sequences but arranged in a different order). A different version of a post-familiarization recognition test includes presenting a pair of sequences and asking participants to choose which sequence in the pair is a word from the artificial language they listened to.

The project was approved by the ethical board of the Basque Center on Cognition, Brain and Language (BCBL), approval received on the 26^th^ of April, 2021, reference number 260421MK.

### Participants

All participants were students from the University of the Basque Country and Murcia University. We recruited Basque-Spanish bilinguals (AoA − age of acquisition − is 2 y.o. for both languages) from the province of Gipuzkoa in the Basque country (N=60, one participant was excluded because he did not show up for the second session). The bilinguals were functioning daily in both languages, the languages were not separated by social domains (e.g., both Basque and Spanish were used interchangeably as a professional, educational and home languages). Participants were equally proficient in both languages (based on the lexical tests administered to all participants in the BCBL database, and the inclusion criterion was that they performed equally well in the lexical and language tests in both languages). Native Spanish monolinguals were recruited in Murcia (N=36) and in the Basque Country (San Sebastian) from those students who had arrived in the Basque Country no more than 4 months before the onset of the experiment (N=44, two additional participants were also tested, but their data was excluded because they did not show up for the second experimental session). In total, we analyzed the data from 59 bilinguals and 78 monolinguals. None of the participants reported any speech/language/hearing disorders. For participation in the experiment, participants received a compensation of 10 Euro. All participants signed a written informed consent form.

### Material

We adapted a classical artificial language learning experiment (Saffran et al., 1996) for the auditory modality, using CV (consonant-vowel) syllables as linguistic speech material (session 1) and non-verbalizable sounds as non-linguistic speech material (session 2). The order of sessions was counterbalanced across participants. As linguistic material, we used 18 syllables arranged into 9 bi-syllabic constituents (referred to as stems further on), each syllable could only be used in one of the constituents. Three other syllables were used to model suffixes (syllables *so, mo*, and *pi*), and three more syllables were used to model prefixes (syllables *fe, po*, and *sa*). The nine stems were divided into three equal groups. In the first group, the three stems could be paired with any of the three prefixes, resulting in nine possible prefixed “words”; in the second group, the three stems could be paired with any of the three suffixes, resulting in nine possible suffixed words; and in the third group, the three stems did not take any affix, resulting in three unaffixed words. For example, a stem *kofa* from the group of suffixed constituents could be used as *kofaso, kofamo*, or *kofapi*. A stem *kani* from the group of prefixed constituents could be used as *fekani, pokani*, or *sekani*. A stem *fumi* from the group of stems that did not receive any affixes was always used as *fumi*.

An important typological distinction between Basque and Spanish, which is relevant to our task, is that Spanish is a prepositional language; while Basque is a postpositional language (functional words are attached to the right). For example, preposition “*in*” in the phrase “in a house” will occur before the noun in Spanish (***en*** casa), and after the noun in Basque (exte***an***). This typological difference might influence the segmentation of a continuous stream of syllables into word-like constituents (de la Cruz-Pavía et al., 2014). Frequent syllables are sometimes interpreted by Basque dominant speakers as postpositions, and by Spanish monolinguals as prepositions in artificial language learning experiments. This determined the need of introducing functional words (i.e., pre/postpositions, articles, interrogative particles) separately from affixes. We added nine “filler” syllables that were inserted between words. The list of all possible stems, affixes and fillers is presented in Table 1.

**Figure 1:**
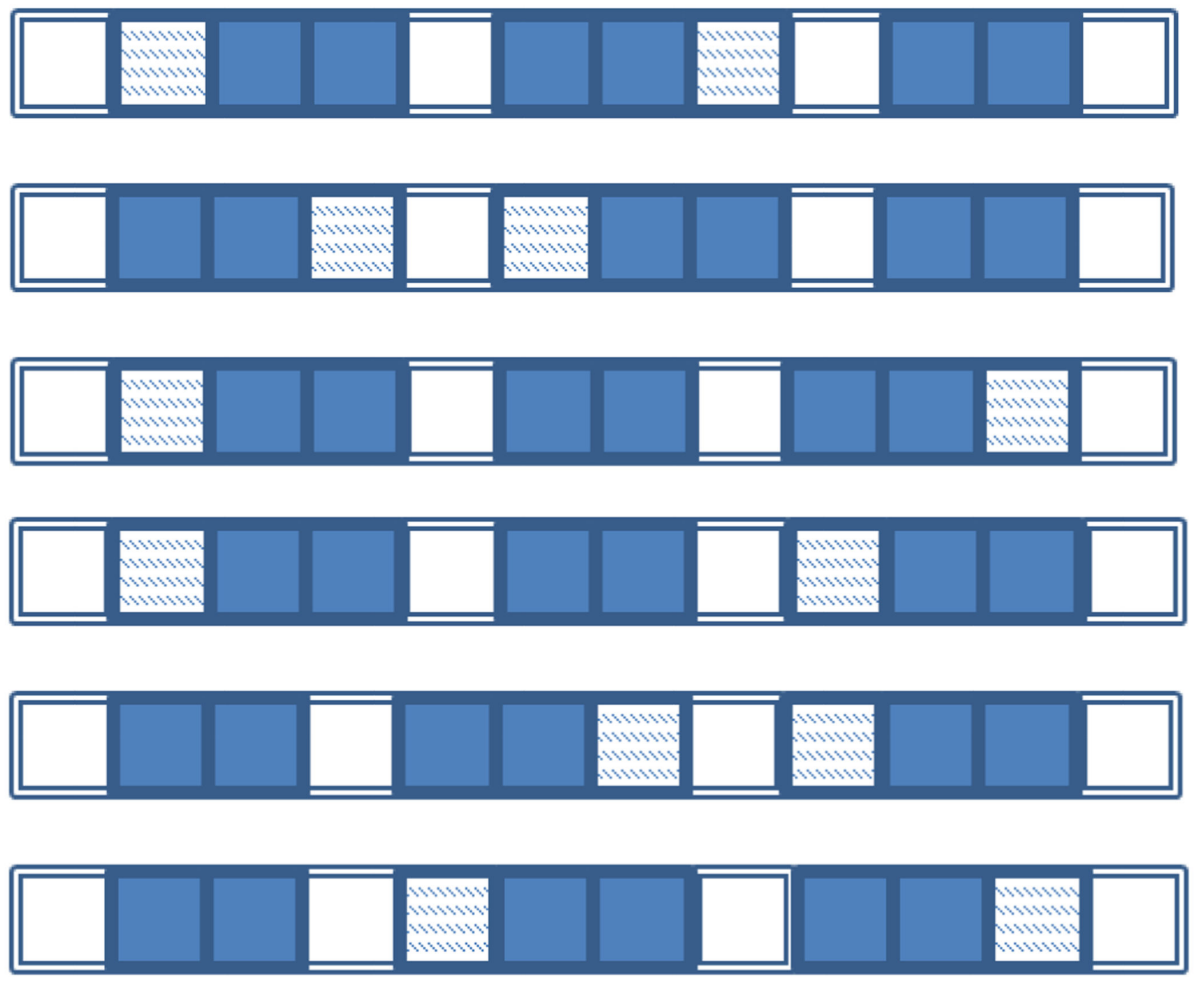
Frames (rows) show the six possible combinations of words, affixes, and fillers. Each square represents a syllable: filled squares represent bi-syllabic stems; patterned squares represent affixes, with suffixes appended after and prefixes before the stem; unfilled squares are fillers (likely to be interpreted as prepositions or postpositions, depending on biases determined by listeners’ native languages). Auditory sequences include six frames, randomly concatenated such that each frame is used equal number of times.

The speech stream (i.e., artificial language) for familiarization exposure was composed of blocks (Figure 1). Each block included six arrangements of three words, in counter-balanced order of prefixed (pref) and suffixed (suff) syllabic sequences and bi-syllabic words (stem-only), making up 8 syllables: (1) pref+suff+stem-only; (2) suff+pref+stem-only; (3) pref+stem-only+suff; (4) suff+pref+stem-only; (5) stem-only+suff+pref; (6) stem-only+pref+suff. The arrangements were randomized within each block. Sixty blocks were created for a complete familiarization stream. In total, each word was embedded into familiarization speech stream 120 times. Each affixed word was used equal number of times with each suffix or prefix. Each filler was used equal number of times to separate the words. These blocks were used to synthesize a continuous familiarization stream in MBROLA (Dutoit et al., 1996), using IT3 (Italian male) voice, with duration of C=100ms and V=140ms, F0=120Hz (monotone). The resulting stream was 15.8 minutes long.

In the resulting stream, the forward transitional probabilities (TPs) between syllables within stems were the highest (100%), between an affix and a stem − intermediate (33%), and between syllables in those syllabic pairs, in which at least one of the syllables was a filler − the lowest (2.8%-11%). Thus, TPs allow for detecting the boundaries between fillers and affixes (lowest TPs), affixes and stems, and between syllables within stems (highest TPs), and discriminating between fillers (modelling functional words) and affixes. The full table of TPs is presented in Table 2.

For the post-familiarization recognition test, we synthesized the tri-syllabic suffixed and prefixed words and bi-syllabic unaffixed stems as separate tokens. For each participant, the same set of three stems was used. We chose one instance of a suffixed stem+suffix (giving three suffixed words as test tokens). That is, if one participant had kofa-so, naku-mo, and sike-pi as suffixed test tokens, another participant could have kofa-mo, naku-pi, and sike-so as suffixed test tokens. Each version of a suffixed word was used equal number of times in the familiarization stream. In the same fashions, we created three unique sets of prefixed words for test tokens.

Additionally, we synthesized three bi-syllabic and six tri-syllabic foils, using the same inventory of syllables used in the familiarization stream. In foils, we combined pairwise those syllables that never co-occurred consecutively in the familiarization stream. For example, a token consisting of a suffix followed by a prefix followed by stem-final syllable is an example of a tri-syllabic foil because in the familiarization stream, a suffix and a prefix syllables never occurred consecutively, and a prefix was never followed by a stem-final syllable. Three sets of possible foils were created and one of the sets was used for each individual participant. The acoustic parameters in the test tokens were the same as in the familiarization stream. Hence, the test tokens were either words (suffixed, prefixed or unaffixed stems) or foils (tri- and bi-syllabic).

As non-linguistic material, we used non-verbalizable noises (sounds of door screaking, footsteps, branch rattling, wind, etc.), which were concatenated into familiarization stream following the same structure as in the linguistic material (one unique sound for one syllable), modeling non-linguistic suffixed and prefixed sequences and fixed bi-syllabic sequences. The stream duration was 19.8 min (it was longer than the linguistic stream because each sound was longer than the CV syllable, but the number of sounds is equal to the number of syllables). Thus, the statistical structure and TPs between sounds were identical to those manifested in linguistic material. Before the sounds were concatenated into a familiarization stream, duration of each sound was equalized to 300ms and then intensity was normalized to 80dB, so that none of the sounds stands out in perceived loudness or length, ensuring that participants could rely solely on statistical (not acoustic) cues to extract discrete and recurrent constituents. Suffixed, prefixed and unaffixed sequences (i.e., non-linguistic words) as well as 3 bi- and 6 tri-syllabic foils were prepared for the post-familiarization recognition test using the same approach employed to prepare the linguistic tokens. A part of the familiarization stream and the test items can be found as audio files in the supplementary material.

### Procedure

The experiment was programmed in PsychoPy and run in the laboratory conditions. The experiment contained two sessions − one on linguistic, and the other on non-linguistic material − in a counter-balanced order across participants. We used explicit instructions. Participants were told that they would listen to an “alien language”, and they would have to detect and memorize the words of this language. Following each type of familiarization, recognition tests were administered.

During the first test, participants listened to a separate token, which was either a word − prefixed, suffixed or unaffixed − or a foil. For each participant a set of three bi-syllabic and six tri-syllabic foils were used. In total, 18 trials were administered. On each trial, participants had to respond whether they thought it was a sequence from the familiarization stream or not.

During the second test, we administered a two-alternative forced-choice test, when participants heard a pair of tokens. One token in each pair was a suffixed word, and the other token was a prefixed word, both were legal constituents used during familiarization equal number of times. We asked participants to choose which token − first or second − was more likely to be a sequence from the familiarization stream. This test aimed to estimate the suffixing versus prefixing preference in bilingual and monolingual samples at the group level. Each suffixed and prefixed constituent was used twice, once in the first position in the pair, and once in the second position in the pair, each time with a different affix, which yielded 6 trials in total.

The procedure for the non-linguistic session was identical. Each session − linguistic and non-linguistic − was approximately 25 minutes in duration.

## Results

### TEST 1: Learnability of suffixed and prefixed sequences in different linguistic populations

In order to be sure that the material − linguistic and non-linguistic − is learnable, we calculated the overall number of correct responses for each participant (accepted stems, suffixed and prefixed sequences and rejected foils) and compared this number with what would be expected by chance (50%, or 9 correct responses out of 18 trials could be given by chance). If morphological properties of native language have no effect on detecting, memorizing and recognition of recurrent word-like constituents in a novel language, we should not see a difference between linguistic populations. Hence, we used Bayesian approach to data analysis, which allows for estimating the strength of support for null hypothesis and use it as evidence of absence of the difference, when the conventional frequentist approaches would only allow stating the absence of evidence that two groups are different. Given that by nature the number of correct responses is an ordinal rather than interval variable, which means that the assumption of normality is likely violated, we applied the Bayesian Mann-Whitney tests with 5 chains of 1000 repetitions and calculated the Bayes factors (BF) using full Cauchy with scaling factor = 0.7 (in Cohens *d* units), which prioritizes neither the null nor the alternative hypotheses (a-priori both hypotheses are equally likely). The analysis was done separately on tests with linguistic and non-linguistic material. All Bayesian tests were run in JASP v. 0.19.1.

**Figure 2:**
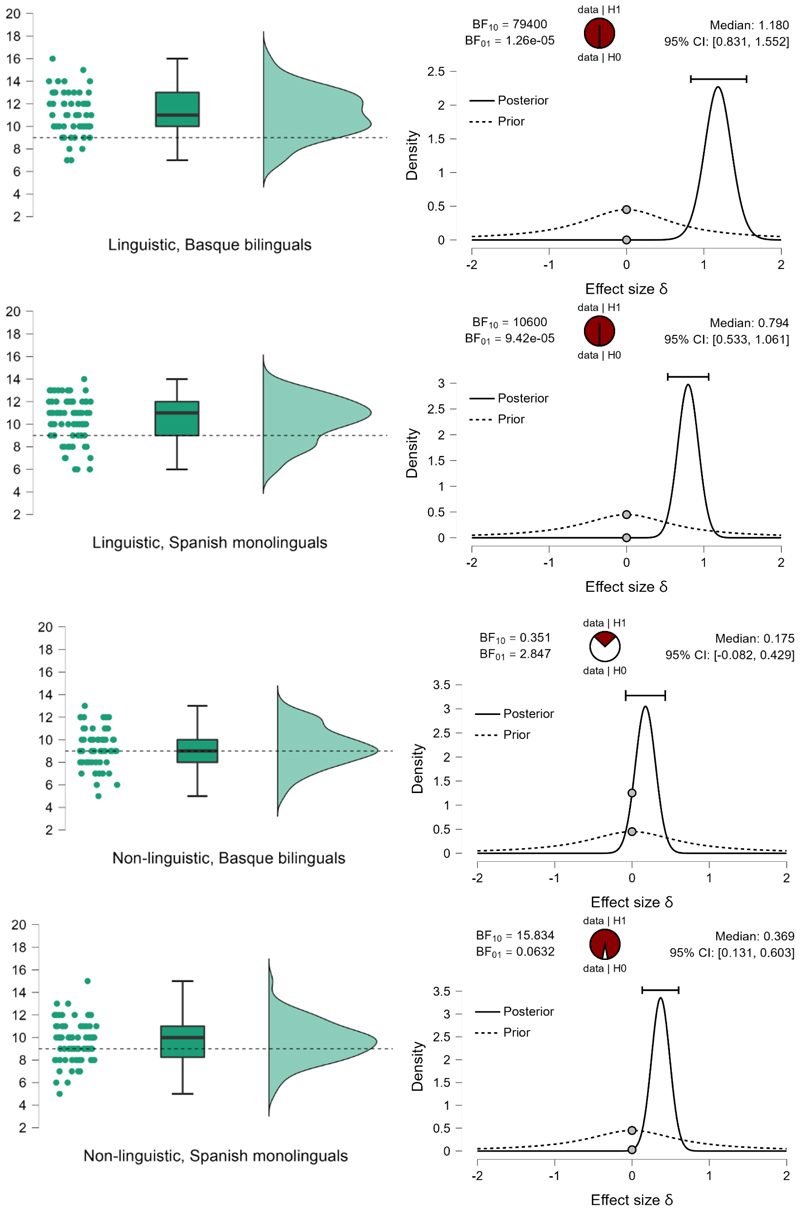
The number of correct responses per group (Basque-Spanish bilinguals and Spanish monolinguals) and material type (linguistic and non-linguistic). The plot in the left column display means and 95% CI. The plot in the middle column displays probability density, individual datapoints, medians, and top and bottom quartiles as whiskers. The dotted line stands for the chance level (50% - 9 correct responses can be given by chance). The plot in the right column shows prior and posterior probabilities (with 95% credible interval) for the difference in the number of correct responses per sample and the average number of correct responses that could be expected by chance. The dots show prior and posterior density at the test value. The pie chart represents the estimated degree of support for the null (H0, unfilled part of the chart) and alternative (H1, filled part of the chart) hypotheses.

Two-tailed tests comparing the number of correct responses with what would be expected by chance (N=9) revealed that linguistic material was processable and sequences were learnable at an above-chance level in both populations: by Basque-Spanish speakers (M=11.1, SE=.26), BF_10_=79,400, and by Spanish monolinguals (M=10.385, SE=.216), BF_10_=10,600, which is decisive evidence that the material was learnt. Non-linguistic material, however, is learnt only by monolinguals (M=9.615, SE=.198), BF_10_=15.834, which is strong evidence that the number of correct responses is above chance. Basque-Spanish bilinguals perform at a chance level (M=9.3, SE=.225), BF_10_=.351, which is moderate but positive evidence in support of the absence of difference from the group-level performance that would be expected by chance. The result pattern is displayed on Figure 2.

During the test, participants had to endorse or reject an item as a word from the alien language, and the test items could be recurrent sequences from the artificial language (i.e., familiarization stream) or foils. That is, correct responses could be *hits* (recurrent sequences endorsed as potential words from the alien language) and *correct rejections* (rejected foils). High performance can rely on efficiency of rejection and accuracy of endorsement. Ordin et al. (2020) showed that endorsement relies on successful retrieval of items from memory. Rejection, on the other hand, relies on detecting the transitions between syllables that violate the regularities embedded into the familiarization stream presented for learning. Given that endorsement and rejection rely on different cognitive mechanisms that have a distinct neural underpinning (Ordin et al., 2020), we decided to use the False Discover Rate (FDR) approach (a common analytic technique in evaluating the efficiency of pattern recognition algorithms and diagnostic tests) to analyze the efficiency of rejection and efficiency of endorsement separately.

Precision (i.e., general accuracy) in recognition test (i.e., the proportion of words among endorsed tokens, or the percentage of correct responses) can be achieved by high *sensitivity*, or *recall* (i.e., proportion of endorsed words among all presented words) and specificity (i.e., proportion of foils that were not endorsed). That is, precision in test is a product of how well people endorse the words and reject the foils. Given the structure of the recognition test, the same level of precision achieved by individuals in two different groups can be the result of high specificity and average recall in one group and average specificity and high level of recall in the second group. Therefore, we compared performance in the recognition test between Basque and monolingual Spanish speakers by focusing on precision, recall and specificity separately. The values on these measures vary between 0 and 1, and this is not dependent on the number of trials presented to each individual. Besides, given that these values are ratios, we convert ordinal variables (number of correct or false responses) into interval variables, which allows for using parametric tests (if the normality assumption is not violated).

We calculated precision, recall and specificity separately on linguistic and on non-linguistic material, and compared these values between Basque-Spanish bilinguals and Spanish monolinguals using Bayesian tests, 2-tailed, full Cauchy’s scaling factor=.707 (see Table 3).

Overall, the data showed that Basque-Spanish bilinguals are better at recognition accuracy (recall, or sensitivity) of embedded constituents compared to Spanish monolinguals. The rejection accuracy, which is based on detecting the violations of transitional probabilities, is not modulated by properties of the native language(s). This can be explained by the fact that the breaks in statistical regularities are universally more salient. Ordin et al. (2020) suggested that violations of statistical structure in acoustic or visual perceptual flow cue environmental changes that require a behavioral response. Faster detection of events that require behavioral response provides individual fitness boost (increase the chances of survival and reproduction), hence the neuro-cognitive mechanisms underlying detection of low TPs is evolutionary stable and is less affected by ontogenetic influences (Polyanskaya et al., 2022). By ontogenetic influences we mean factors that might affect the development of individuals, including factors pertaining to properties of the ambient language. Detecting recurrent constituents in the environment is a by-product of a more ancient mechanisms that have been honed for tracking breaches in statistical congruency (i.e., troughs in the TPs), and is more easily modulated by individual experiences. That is why we see the effect of the native language on recall (endorsement efficiency), but not on specificity (rejection accuracy).

**Figure 3:**
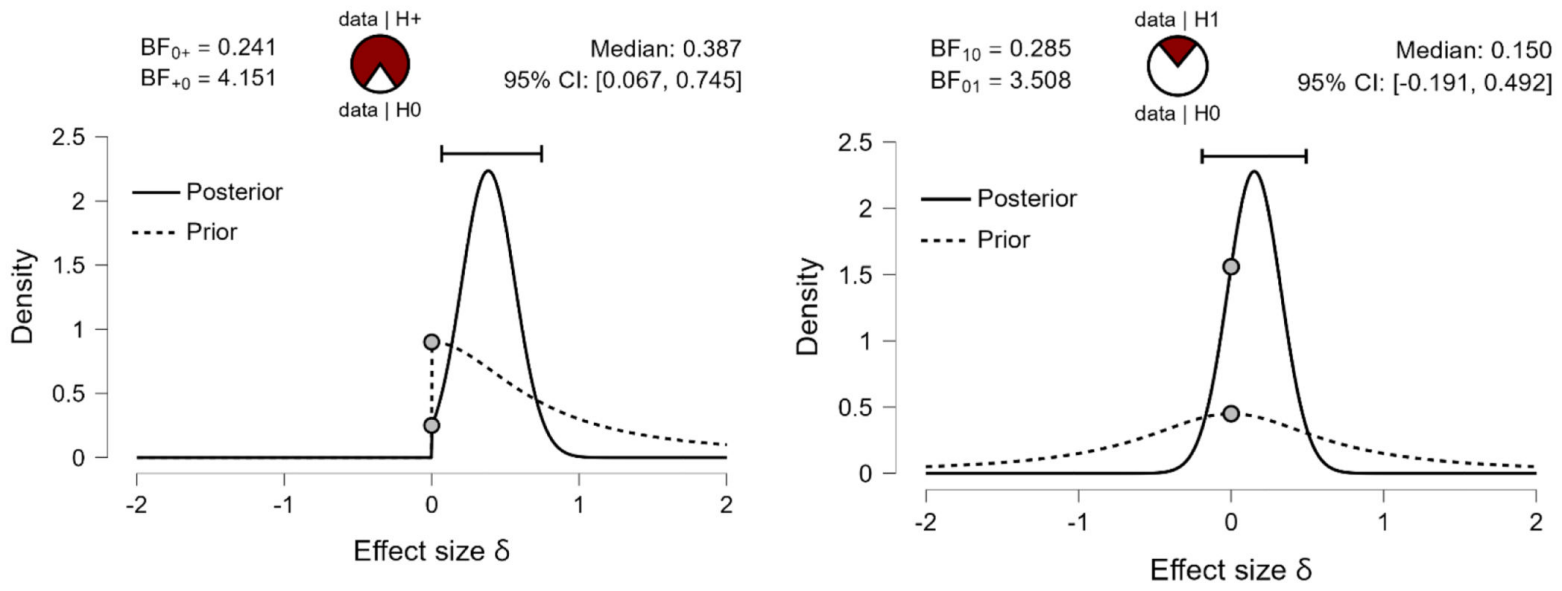
Prior and posterior probabilities (with 95% credible interval) and strength of evidence for the alternative and the null hypotheses given the observed data. **Left:** Basque-Spanish bilinguals recognize prefixed sequences better than Spanish monolinguals (the alternative hypothesis is 4 times more likely than the null hypothesis − Basque bilinguals do not recognize prefixed sequences better than Spanish monolinguals). **Right:** Basque-Spanish bilinguals and Spanish monolinguals recognize suffixed sequences equally likely (the null hypothesis is 3.5 times more likely than the alternative hypothesis − there is difference in recognition rate of prefixed suffixes between the groups). The pie charts represent the estimated degree of support for the null (H0, unfilled part of the chart) and alternative (H1, filled part of the chart) hypotheses.

I suggest that the improved *recall* in the group of Basque-Spanish bilinguals compared to Spanish monolinguals is accounted for by enhanced experience with the prefixed words in the former group (the Basque language makes use of inflectional prefixes, the Spanish language has no inflectional prefixes). To ensure that the enhanced *recall* is driven by better recognition of prefixed linguistic sequences, we compared the percentage of endorsed prefixes sequences (*hits_prefixed*) and endorsed suffixed sequences (*hits_suffixed*) by Basque-Spanish bilinguals and Spanish monolinguals using Bayesian independent samples Mann-Whitney tests (full Cauchy with scaling factor=.707, both the alternative and the null hypotheses are equally likely, 5 chains of 1000 repetitions and repeatability seed 10, to enhance the test robustness). We predicted that Basque-Spanish bilinguals, due to their experience with grammatical prefixes, will endorse more prefixed sequences on linguistic material than Spanish monolinguals, hence the testis 1-tailed. Given the data (M=76.8% of presented prefixed sequences are endorsed by Basque-Spanish bilinguals, SE=3.15, and 62.8% of presented prefixed sequences are endorsed by Spanish monolinguals, SE=3.55), the alternative hypothesis (that Basque-Spanish bilinguals endorse more prefixed sequences) is 4 times more likely than the null hypothesis, BF_10_=4.151. For the rate of endorsement of the suffixed sequences (2-tailed, because we test the hypothesis that endorsement rate on suffixed sequences is different, without specifying the direction of difference), the zero hypothesis is 3.5 times more likely, BF_10_=.285. The analysis showed that Basque bilinguals indeed recognize prefixed sequences better than Spanish monolinguals, and suffixed sequences are recognized equally well in both groups. This result pattern is shown on Figure 3.

### TEST 2: Prefix preference

In recognition test 2, participants had to listen to a pair of sequences − one prefixed and one suffixed, both of which had occurred in the familiarization stream an equal number of times − and selected which one they thought was more likely to be a word from the alien language they listened to. As both responses are correct, test 2 probes participants’ preference for an affix appended at the end (suffix) vs. at the beginning (prefix) of the stem.

First, we ran Bayesian Mann-Whitney one-sample two-tailed tests (full Cauchy, scaling factor=.707, 5 chains of 1000 repetitions) to compare the number of preferred prefixed sequences with what would be expected by chance (50%). Given that data, Basque-Spanish speakers selected linguistic prefixed sequences (M=47.74%, SE=2.149) at a rate that is not different from what would be expected by chance (BF_10_=243, the null hypothesis is over 4 times more likely than the alternative). Spanish monolingual speakers (M=41.88%, SE=2.21) select linguistic prefixed sequences at a rate that is lower than what would be expected by chance (BF_10_=68.86, providing decisive evidence for the hypothesis that the rate of endorsement is different from 50% chance level). Direct comparison between groups, however, provides ambiguous results (BF_10_=1.153, suggesting that, given the data, there is almost equal evidence to support the null and the alternative hypothesis). The data cannot confirm that the preference for suffixed over prefixed sequences in the group of Spanish monolinguals and the lack of this preference in the groups of Basque-Spanish bilinguals does not provide sufficient evidence that Basque speakers, on average, select more prefixed sequences. I suggest that the lack of preference in the group of bilinguals lead to higher volatility in answers at the individual level, which obscures the group-level differences that would be expected if one group exhibits a suffixing preference, and the other group − a prefixing preference.

On non-linguistic material the data supports the hypothesis that neither Basque-Spanish bilinguals (M=49.43%, SE=2.89) nor Spanish monolinguals (M=50.0%, SE=2.375) revealed a suffix-over-prefix (or reverse) preference (BF_10_=.148 for Basque speakers and BF_10_=.132 for Spanish monolingual speakers, providing a very strong evidence for the null and showing that, given the data, the null hypothesis is 6.8 times more likely in the former group, and 7.57 times more likely in the latter group).

These results suggest a modulatory effect of the native language on the suffixing bias on linguistic material. Bilinguals accept both linguistic prefixed and suffixed sequences at an equal rate, while monolinguals show a stronger preference for suffixed sequences (and consequently do not select prefixed sequences). The result pattern is displayed on Figure 4a (bilinguals) and 4b (monolinguals).

### Results Overview

Overall, the analysis of both tests shows that better recall in the recognition test on linguistic material in the group of Basque bilinguals is driven by better recognition and endorsement rate of prefixed sequences, which is probably driven by familiarity with inflectional prefixes in their native language. The effect is not observed on non-linguistic material, suggesting that the strategies tuned for processing peculiarities of native language morphology are not transferred from linguistic to non-linguistic domain. Also, we observed a preference for suffixed over prefixed linguistic sequences in the group of Spanish monolinguals, which is not transferred to non-linguistic material either. Experience with the Basque language that utilizes both suffixes and prefixes overrides the suffixing preference, so that Basque-Spanish bilinguals exhibit neither prefixing nor suffixing preferences. Taken together, this pattern indicates that the typological suffixing bias is probably restricted to language and it is the properties of the ambient language rather than properties of the domain-general cognitive systems that lead to the emergence of cognitive bias in processing of speech-like perceptual input.

## Discussion

In the current study, we attempted to address the question regarding the origin of the suffixing bias. The term suffixing bias in this sense refers to typological distribution of world’s languages in the spectrum from strongly suffixing to strongly prefixing, not to cognitive bias. However, cognitive bias − constraints on learning, perception, memory, attention, and articulatory movements that make the beginning of the auditory sequences more salient and more easily memorized − might shape the language structures by disfavoring variable parts (i.e., prefixes) at the word onsets. In other words, cognitive domain-general suffixing bias might be the origin of the typological bias in distribution of affixation language properties. Alternatively, suffixing bias be a domain-specific phenomenon restricted only to language, and draw on peculiarities pertaining to speech processing rather than on domain-general memory, learning and perception mechanisms. To address the question of the suffixing bias origin, we used statistical learning experiments, in which embedded words were either suffixed, or prefixed, or unaffixed. The artificial languages were composed of either linguistic material (syllables) or non-linguistic material (noises). The experiment had two post-familiarization tests: preference test, when participants had to choose between a prefixed and a suffixed words from an artificial language, and a test, in which participants had to listen to an acoustic sequence (syllables for linguistic material and noises for non-linguistic material, which was either a recurrent sequence that occurred multiple times during the learning stage, or a foil) and to report whether it was a word from a artificial language or not. The experiment was run in the population of Spanish monolinguals (Spanish uses only suffixes to express grammatical meanings) and in the population of Basque bilinguals (Basque uses both suffixes and prefixes to express grammatical meanings, hence Basque-Spanish bilinguals have more experience with processing prefixes than Spanish monolinguals).

The data shows that the preference for suffixed over prefixed linguistic sequences is modulated by the presence of grammatical prefixes in the native language: if the native language of an individual uses both prefixes and suffixes to express grammatical functions, then individuals manifest no preference for suffixed vs. prefixed sequences in a recognition test in a Saffran-style artificial language learning experiment. Although we showed it for one pair of languages (Basque and Spanish), this conclusion agrees with the conclusion of Martin and Culbertson (2020), who used English and a heavily prefixing Bantu language Kîîtharaka. Martin and Culbertson (2020) showed that if the native language of an individual manifests grammatical functions solely by prefixes, then the suffixing bias is overturned, and instead a prefixing preference can emerge. However, contrary to previous studies, we did not observe any influence of typological properties in the native language morphology on the preference for suffixed or prefixed non-linguistic sequences, suggesting that the effect is limited to the linguistic domain. Probably, the difference in result patterns across studies can be accounted for by different methodological approaches. Earlier empirical results were based on similarity judgements (e.g., people had to judge whether ***“to-ta-be”*** or ***“be-to-ta”*** is more similar to ***“to-ta”***) or another task, in which the sequences − linguistic and non-linguistic − are presented in isolation. However, statistical learning experiments include detecting the boundaries between discrete constituents in a continuous sensory input, extracting these sequences, memory encoding (committing the extracted sequences to memory) and decoding (retrieval of the memorized sequences from memory) during the recognition test. The underlying cognitive processes are different from those implicated during similarity judgement. The typological properties of an ambient language can exercise an effect on a single cognitive mechanism or on a limited set of such mechanisms that are implicated by task execution, while an ability relying on a large set of cognitive mechanisms (e.g., statistical learning) is more robust to the influence of task-irrelevant influences. That is why similarity judgement of non-linguistic sequences is affected by native language properties to the greater degree than performance in statistical learning on non-linguistic material.

As a multi-faceted ability that relies on a whole set of cognitive mechanisms, statistical learning is evolutionary ancient and emerged for non-linguistic purposes, including the need to structure environmental sensory input and build internal models of the worlds (Badcock et al., 2019; Friston, 2005). This ability was then recycled for speech processing in the *homo* genus. Should the suffixing bias be linked to the general-purpose mechanisms, then we would have observed it on non-linguistic material. As the preference can only be observed on linguistic stimuli and it is interacting with the typological properties of the ambient language, the phenomenon is probably limited to language domain, and is not a result of evolutionary adaptation of language code to pre-installed cognitive machinery.

We also found that overall, Basque speakers perform better than Spanish monolinguals, and higher precision is explained by better recognition of prefixed sequences in the former group. Individuals in both groups reject foils efficiently. Detection of statistical incongruencies is a more fundamental product of tracking statistical regularities that functions in any type of environment (Ordin et al., 2021; 2022), thus it is more robust to environmental differences, including differences in linguistic environment, and we did not observe any difference between groups in specificity. Recall, or recognition of statistically congruent tokens, by contrast, is more subject to the effect of native language because linguistic constituents are defined differently across languages, both grammatically and prosodically. Statistical learning is used for structuring a continuous speech flow and building internal models of the recurrent constituents (Badcock et al., 2019), with further cognitive processing happening on these internal models, which may differ depending on individual experience with differential sets of grammatical and prosodic cues.

The results can potentially be accounted for bilingual advantage in language domain in one the groups. Although the existence of bilingual advantage is statistical learning is still debated, with evidence both for and against it (Weiss et al., 2019 for review), a series of previous studies involving Basque-Spanish bilinguals vs. Spanish monolinguals did not reveal significant differences between populations (Aguasvivas et al., 2024; Ordin et al., 2017; Ordin et al., 2020; Polyanskaya et al., 2022). Good and bad learners are distributed equally in both populations, and when the samples are collected randomly, so that each individual has an equal chance to be included into the sample, no differences between bilingual and monolingual groups is observed in performance on the recognition test. As in earlier artificial language learning studies, in which the morphological properties were not modelled, the differences between bilingual and monolingual populations under study were not observed at cognitive level, we assume that the difference in this task is explained by the effect of the presence of inflectional prefixes in Basque. We cannot exclude the possibility that bilinguals are more attuned to novel language features due to the need to handle multiple languages and thus have heightened awareness of novel language features, but at the same time Basque-Spanish bilinguals did not have to treat prefixes as novel linguistic properties because they were in one of their native languages (Basque), therefore it is more likely that the effect is explained by their previous experience with this feature via acquisition of Basque rather than by heightened linguistic awareness due to bilingualism per se.

It is important to mention two limitations of the study. First, our theoretical assumption is based on the reality of the typological suffixing bias. However, it is important to emphasize that many typological biases are not held true after controlling for the genealogical relations or for the confounds related to geographical distribution of languages. For example, a long-established bias that OV order precludes the possibility of prefixing inflexions, while VO allows both suffixing and prefixing (Bybee et al.,1990; Dryer, 1992; 2011; Song, 2012) seems less convincing once genealogical and areal potential confounds are controlled for (Guzmán et al., 2022). The observed tendency across world’s languages to express grammatical meanings by suffixes can be a result of rapid vertical (cross-generation adaptation and diachronic development) and/or horizontal transfer (e.g., language contacts) of a preference that emerged in a particular population and then spread across geographical regions by means of social learning.

The second limitation is related to the fact that the artificial language has no semantics. Potentially, suffixes and prefixes may play different roles in statistical learning when learning a language where constituents are mapping to meaning (Hoppe et al., 2020; St. Clair et al., 2009; Vujović et al., 2021). In a series of simulations and experiments, including artificial language learning, it was shown that suffixes facilitated categorization of artificial language units into classes (similar to splitting the lexical units into grammatical categories), and prefixes facilitated learning of stems following the prefix (Hoppe et al., 2020; St. Clair et al., 2009). A large-scale hypothesis-driven study (N=434) did not replicate earlier findings in a straightforward way (Vujović et al., 2021). The differences between functional load of prefixes and suffixes are more subtle and modulated by frequency of cues used for processing: participants in a suffix condition were better able discriminate between frequent, but uninformative cues and low-frequency, informative cues which led to different patterns of generalization. However, the current finding suggests that dividing morphologically complex constituents into stems and affixes is possible based on the relative frequency of the composing syllables and their co-occurrences, without recourse to semantic meaning. While there might be some additional differences between prefixing and suffixing once you bring in a reference world (Hoppe et al., 2020; Vujović et al., 2021), the current study shows that some differences can be observed even without this.

In sum, the preference for suffixes or prefixes is modulated by the morphology of the native language and is only observed on linguistic material. Accuracy in discrimination between prefixed words that recurrently occurred in the familiarization stream and the foils is higher in Basque-Spanish bilinguals (who have experience with processing grammatical prefixes) than in Spanish monolinguals (who do not have this experience); this between-group difference in only evident on linguistic material and draws on better recall (proportion of accepted words to the total number of all presented words, or endorsement accuracy), not on differences between groups in specificity (proportion of rejected foils to the total number of all presented foils, or rejection accuracy). We argued that specificity is affected by natural selection pressure and it is the same for suffixed and prefixed sequences on all types of material and in both investigated populations. Recall is subject to the influence of native language in the course of individual development (more exposure to prefixes results in better recall of prefixed sequences), but this effect is constrained to linguistic material. As an overall conclusion, we found no support for pre-linguistic domain-general preference for suffixed sequences. Our results provide some evidence in favor of a linguistic origin of the suffixing bias. The suffixing preference of individuals within a particular linguistic community can be modulated by the typological properties of the ambient language in this community, or position of the native language in the spectrum from suffixing to prefixing languages.

## Figures and Tables

**Figure 4a:**
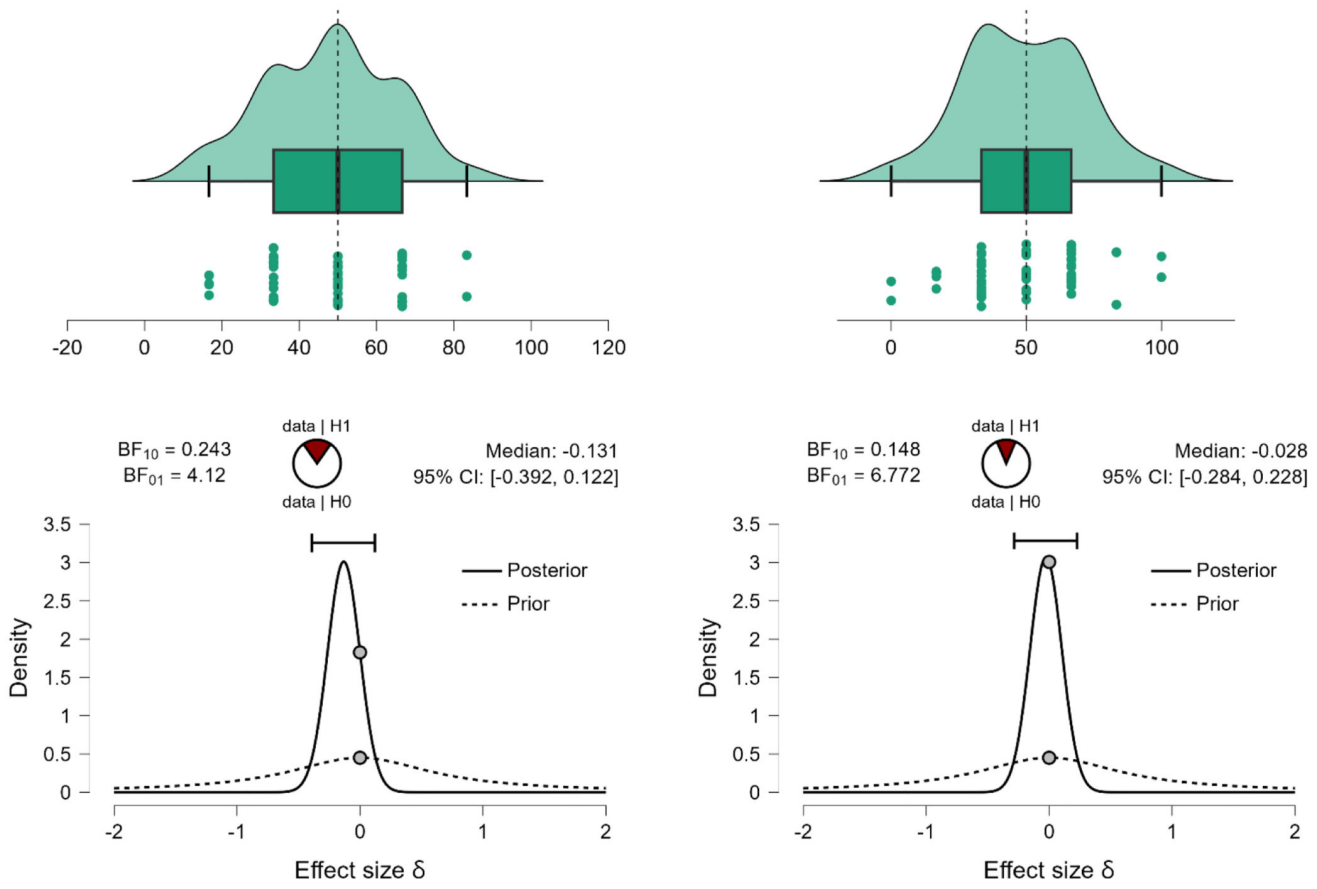
The percentage of prefixed sequences (preferred over suffixed sequences) on linguistic (left) and non-linguistic (right) material by Basque-Spanish bilinguals. The upper plots display probability density, individual datapoints, medians, top and bottom quartiles as whiskers. The bottom plots show prior and posterior probabilities (with 95% credible interval) for the difference in the number of selected prefixed sequences by participants and the number that could be expected by chance. The dots show prior and posterior density at the test value. The pie chart represents the estimated degree of support for the null (H0, unfilled part of the chart) and alternative (H1, filled part of the chart) hypotheses.

**Figure 4b:**
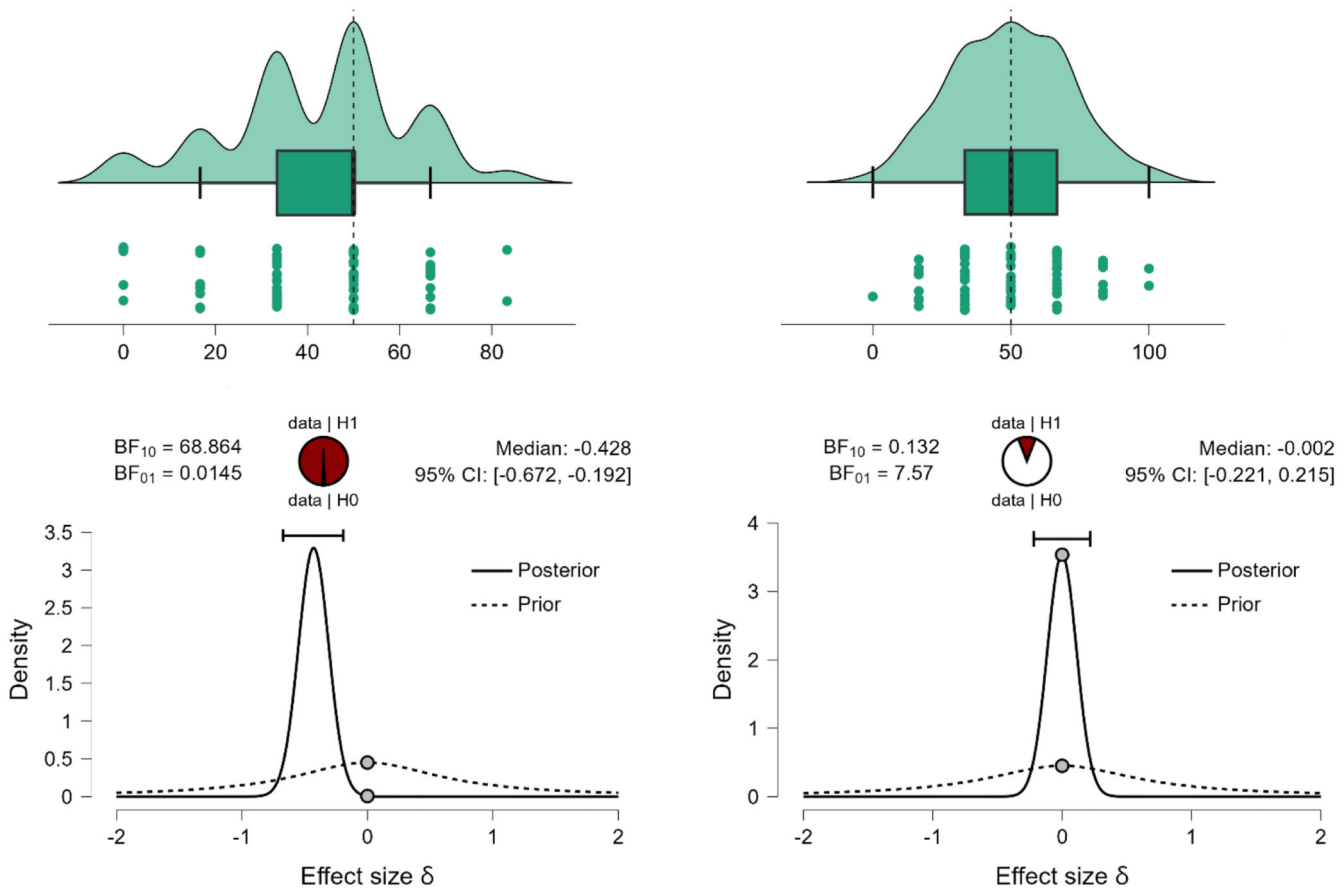
The percentage of prefixed sequences (preferred over suffixed sequences) on linguistic (left) and non-linguistic (right) material by Spanish monolinguals. The upper plots display probability density, individual datapoints, medians, top and bottom quartiles as whiskers. The bottom plots show prior and posterior probabilities (with 95% credible interval) for the difference in the number of selected prefixed sequences by participants and the number that could be expected by chance. The dots show prior and posterior density at the test value. The pie chart represents the estimated degree of support for the null (H0, unfilled part of the chart) and alternative (H1, filled part of the chart) hypotheses.

**Table 1:** The list of prefixes, suffixes, stems and fillers used in the linguistic material. Each suffixed stem was used with each suffix, hence providing an equal number of occurrences of KOFA-SO, KOFA-MO, KOFA-PI, NAKU-SO, NAKU-MO, etc. The same is applied to prefixed stems and prefixes.

Prefixes	Prefixed stems	Suffixed stems	Suffixes	Unaffixed stems	Fillers
fe	kani	Kofa	so	Fumi	ma
po	mupe	naku	mo	nupa	fi
sa	nosu	sike	pi	mefo	pu
					sho
					se
					shu
					ne
					ki
					sha

**Table 2:** The transitional probabilities between syllables in different syllabic pairs.

Transition from	Transition to	Transitional probability	Comments
Stem-initial	Stem-final	1 (100%)	Within stems, the first syllable predicts the second with 100% probability
Stem-final	Suffix	1/3 (33%)	A stem-final syllable in a suffixed word predicts that the next syllable will be a suffix, and there are three possible suffixes in the inventory, all used equal number of times with each stem.
Stem-final	Filler	1/9 (11%)	A stem-final syllable in a word that is never used with affixes, the stem-final syllable predicts that the next syllable is a filler, and there are nine possible fillers, all counter-balanced in positions.
Prefix	Stem-initial	1/3 (33%)	Each prefix is attached to each of the three possible prefixed words; hence the prefix can predict the next syllable with 33% probability
Suffix	Filler	1/9 (11%)	A suffix can predict that the following syllable is one of the nine fillers.
Filler	Filler	1/36 (2.8%)	A filler can predict that the next syllable will be another filler, or prefix, or a stem-initial syllable of an unaffixed word, or a stem-initial syllable of a suffixed word. Each of these cases can occur with equal probability (1/4, or 25%). A number of possible prefixes id three, each prefix can occur with equal probability, giving TPs between filler and prefix 1/12 (or 8.3%). The same calculation is applied to other transitions.
Filler	Prefix	1/12 (8.3%)	
Filler	Stem-initial of a suffixed word	1/12 (8.3%)	
Filler	Stem-initial of an unaffixed word	1/12 (8.3%)	

**Table 3:** Comparing Precision, Recall and Specificity on linguistic and non-linguistic material between Basque and monolingual Spanish participants, on linguistic and non-linguistic material.

		Mean (SE of Mean)	BF_10_	Test result and interpretation
**linguistic**	**Precision**	bilinguals: M=.605 (.014) monolinguals: M=.564 (.013)	1.3	The likelihood that Basque-Spanish bilinguals exhibit higher precision than Spanish monolinguals is only 1.3 higher. This strength of evidence, in Jeffrey’s term, is anecdotal and barely worth mentioning for the current dataset, although useable for adjusting the priors in the following tests).
	**Recall**	bilinguals: M=.742 (.022) monolinguals: M=.638 (.025)	11.305	Basque-Spanish bilinguals, as a group, are 11 times more likely to recognize the recurrent sequences from the familiarization stream compared to Spanish monolinguals, which is very strong evidence that *recall* is higher in the groups of individuals who have experience with prefixes in at least one of their native languages compared to those individuals who have no grammatical prefixes in their native languages.
	**Specificity**	bilinguals: M=.492 (.028) monolinguals: M=.516 (.02)	.234	There is substantial evidence in favor of the null hypothesis, namely, that Basque-Spanish bilinguals and Spanish monolinguals do not differ in rejection accuracy (foils are rejected equally well by individuals from both experimental groups): the null hypothesis is 4.3 times more likely than the alternative hypothesis.
**non-linguistic**	**Precision**	bilinguals: M=.511 (.01) monolinguals: M=.523 (.008)	.285	There is substantial evidence in favor of the null hypothesis, namely, that Basque-Spanish bilinguals and Spanish monolinguals do not differ in overall accuracy (precision) on non-linguistic material. The null hypothesis is 3.5 times more likely than the alternative.
	**Recall**	bilinguals: M=.693 (.022) monolinguals: M=.749 (.017)	1.244	The likelihood that Spanish monolinguals exhibit higher precision than Basque-Spanish monolinguals is only 1.2 higher. This strength of evidence, in Jeffrey’s term, is anecdotal and barely worth mentioning.
	**Specificity**	bilinguals: M=.341 (.02) monolinguals: M=.319 (.016)	.257	There is substantial evidence in favor of the null hypothesis, namely, that Basque-Spanish bilinguals and Spanish monolinguals do not differ in rejection accuracy (foils are rejected equally well by individuals from both experimental groups): the null hypothesis is 3.9 times more likely than the alternative hypothesis

## Social Media Summary

We discuss why we say *walk****ed*** and not ***ed****walk* to speak about the past, or *pen****s*** and not ***s****pen* to talk about multiple objects

## Footnotes

i Circumfix should not be confused with a combination of a prefix and suffix added to the same root (e.g., German word ***ge***mach***t***, in which ***ge-*** and ***-t*** added to the root *mach* express the same purpose to build a participle). If a morphological element before the root and after the root serve to express the same (grammatical) meaning (e.g., person, tense, case, number, etc), and one element cannot be added to the root without adding the other part, then the whole two-element morpheme is referred to as a circumfix. A prefix does not require a suffix, and a suffix does not require a prefix. Besides, prefix and affix each expresses a separate grammatical meaning (e.g., in the Basque word ***ga***bil***tza***, from the verb *ibili − to walk*, with the root *bil*, a prefix expresses a person − first person − and suffix expresses number − plural; suffix -***tza*** can be combined with other prefixes, e.g., zabiltza, dabiltza, and prefix ***da-*** can be combined with other suffixes or used without any suffix, e.g., ***da***bil, ***da***bil***kit***, etc.)

## References

[R1] Aguasvivas J, Cespón J, Carreiras M (2024). Does bilingual experience influence statistical language learning?. Cognition.

[R2] Antinucci F, Duranti A, Gebert L (1979). Relative clause structure, relative clause perception, and the change from SOV to SVO. Cognition.

[R3] Bybee J, Pagliuca W, Perkin RD, Croft W, Kemmer S, Keith D (1990). Studies in typology and diachrony Papers presented to Joseph H Greenberg on his 75th birthday.

[R4] Badcock PB, Friston KJ, Ramstead MJD, Ploeger A, Hohwy J (2019). The hierarchically mechanistic mind: an evolutionary systems theory of the human brain, cognition, and behavior. Cognitive, Affective and Behavioral Neuroscience.

[R5] Blevins J (2004). Evolutionary phonology: The emergence of sound patterns.

[R6] Bloom LC (2006). Two-component theory of the suffix effect: Contrary evidence. Memory & Cognition.

[R7] Bruening PR, Brooks PJ, Alfieri L, Kempe V, Dabašinskienė I (2012). Children’s Tolerance of Word-Form Variation. Child Development Research.

[R8] Christiansen MH, Chater N (2001). Connectionist psycholinguistics: Capturing the empirical data. Trends in Cognitive Science.

[R9] Clark EV, Gelman SA, Byrnes JP (1991). Perspectives on language and thought: Interrelations in development.

[R10] Clark EV, Spencer A, Zwicky AM (1998). The handbook of morphology.

[R11] Conway CM (2020). How does the brain learn environmental structure? Ten core principles for understanding the neurocognitive mechanisms of statistical learning. Neuroscience & Biobehavioral Reviews.

[R12] Croft W (2001). Radical construction grammar.

[R13] Cutler A, Hawkins JA, Gilligan G (1985). The suffixing preference: A processing explanation. Linguistics.

[R14] De la Cruz-Pavía I, Elordieta G, Sebastián-Gallés N, Laka I (2014). On the role of frequency-based cues in the segmentation strategies of adult OV/VO bilinguals. International Journal of Bilingual Education and Bilingualism.

[R15] Dienes A, Altmann GTM, Gao S (1999). Mapping across domains without feedback: A neural network model of transfer of implicit knowledge. Cognitive Science.

[R16] Dryer MS, Haspelmath M, Dryer MS, Gil D, Comrie B (2005). The world atlas of language structures.

[R17] Dryer MS (1992). The Greenbergian word order correlations. Language.

[R18] Dryer MS (2011). The evidence for word order correlations. Linguistic Typology.

[R19] Enrique-Arias A (2002). Accounting for the position of verbal agreement morphology with psycholinguistic and diachronic explanatory factors. Studies in Language.

[R20] Erdeljac V, Mildner V (1999). Temporal structure of spoken-word recognition in Croatian in light of the cohort theory. Brain and Language.

[R21] Farley LA, Neath I, Allbritton DW (2007). Irrelevant speech effects and sequence learning. Memory & Cognition.

[R22] Friston KJ (2005). A theory of cortical responses. Philosophical Transactions of the Royal Society of London B: Biological Sciences.

[R23] Gasser M (1994). Acquiring receptive morphology: A connectionist model.

[R24] Gibson E, Marantz A, Miyashita Y, O’Neil W (2000). Image, language, brain.

[R25] Gleitman L, Papafragou A, Holyoak KJ, Morrison RG (2005). The Cambridge handbook of thinking and reasoning.

[R26] Greenberg JH, Greenberg JH (1957). Essays in linguistics.

[R27] Grosu A, Thompson S (1977). Constraints on the distribution of NP clauses. Language.

[R28] Guzmán Naranjo M, Becker L (2022). Statistical bias control in typology. Linguistic Typology.

[R29] Hall G (1991). Perceptual and associative learning.

[R30] Hammarström H (2021). Measuring Prefixation and Suffixation in the Languages of the World.

[R31] Hawkins JA (1983). Word Order Universals.

[R32] Hawkins JA, Cutler A, Hawkins JA (1988). Explaining language universals.

[R33] Hawkins JA, Gilligan G (1988). Prefixing and suffixing universals in relation to basic word order. Lingua.

[R34] Hoppe DB, Rij J, Hendriks P, Ramscar M (2020). Order Matters! Influences of Linear Order on Linguistic Category Learning. Cognitive Science.

[R35] Hupp J, Sloutsky V, Culicover P (2014). Evidence for a domain-general mechanism underlying the suffixation preference in language. Language and Cognitive Processes.

[R36] Kersten AW, Goldstone RL, Schaffert A (1998). Two competing attentional mechanisms in category learning. Journal of Experimental Psychology: Learning, Memory, and Cognition.

[R37] Kikuchi Y, Sedley W, Griffiths TD, Petkov C (2018). Evolutionarily conserved neural signatures involved in sequencing predictions and their relevance for language. Current Opinions in Behavioral Sciences.

[R38] Lewis RL, Vasishth S, Van Dyke JA (2006). Computational principles of working memory in sentence comprehension. Trends in Cognitive Sciences.

[R39] Mackintosh NJ (1975). A theory of attention: Variations in the associability of stimuli with reinforcement. Psychological Review.

[R40] Marcus GF, Fernandes KJ, Johnson SP (2007). Infant rule learning facilitated by speech. Psychological Science.

[R41] Marslen-Wilson W (1987). Functional parallelism in spoken word recognition. Cognition.

[R42] Martin A, Culbertson J (2020). Revisiting the suffixing preference: native-language affixation patterns influence perception of sequences. Psychological Science.

[R43] Milne AE, Petkov CI, Wilson B (2018). Auditory and visual sequence learning in humans and monkeys using an artificial grammar learning paradigm. Neuroscience.

[R44] Morton J (1976). Two mechanisms in the stimulus suffix effect. Memory & Cognition.

[R45] Neath I (1993). Distinctiveness and serial position effects in recognition. Memory and Cognition.

[R46] Ordin M, Polyanskaya L, Samuel AG (2021). An evolutionary account of intermodality differences in statistical learning. Annals of the New York Academy of Sciences.

[R47] Ordin M, Polyanskaya L, Soto D (2020). Neural bases of learning and recognition of statistical regularities. Annals of the New York Academy of Sciences.

[R48] Parmentier FBR, Tremblay S, Jones DM (2004). Exploring the suffix effect in serial visuospatial short-term memory. Psychonomic Bulletin & Review.

[R49] Repp BH (1992). Probing the cognitive representation of musical time: Structural constraints on the perception of timing perturbations. Cognition.

[R50] Rodd JM (2004). When do leotards get their spots? Semantic activation of lexical neighbors in visual word recognition. Psychonomic Bulletin and Review.

[R51] Saffran JR, Aslin RN, Newport EL (1996). Statistical Learning by 8-Month-Old Infants. Science.

[R52] Sapir E (1921). Language.

[R53] Saygin AP, Dick F, Wilson SW, Dronkers NF, Bates E (2003). Neural resources for processing language and environmental sounds: Evidence from aphasia. Brain.

[R54] Smith LB, Jones SS, Landau B, Gershkoff-Stowe L, Samuelson L (2002). Object name learning provides on-the-job training for attention. Psychological Science.

[R55] St Clair MC, Monaghan P, Ramscar M (2009). Relationships Between Language Structure and Language Learning: The Suffixing Preference and Grammatical Categorization. Cognitive Science.

[R56] Vujović M, Ramscar M, Wonnacott E (2021). Language learning as uncertainty reduction: The role of prediction error in linguistic generalization and item-learning. Journal of Memory and Language.

[R57] Weiss DJ, Schwob N, Lebkuecher AL (2019). Bilingualism and statistical learning: Lessons from studies using artificial languages. Bilingualism: Language and Cognition.

